# Boundary-driven delayed-feedback control of spatiotemporal dynamics in excitable media

**Published:** 2025-12-01

**Authors:** Sebastián Echeverría-Alar, Wouter-Jan Rappel

**Affiliations:** Department of Physics, University of California, San Diego, California 92093, USA

## Abstract

Scroll-wave instabilities in excitable domains are central to life-threatening arrhythmias, yet practical methods to stabilize these dynamics remain limited. Here, we investigate the effects of boundary layer heterogeneities in the spatiotemporal dynamics of a quasi-2D semidiscrete excitable model. We reveal that a novel boundary-driven mechanism suppresses meandering and chaotic spiral dynamics. We show how the strength of the heterogeneities mediates the emergence of this regulation through a pinning-unpinning-like transition. We derive a reduced 2D model and find that a decrease in bulk excitability and a boundary-driven delayed-feedback underlie the stabilization. Our results may point to alternative methods to control arrhythmias.

Spatially extended nonlinear systems can exhibit complex spatiotemporal dynamics, often after undergoing an instability [1, 2]. Examples include signaling protein waves on cell membranes [3], oscillations in chemical reactions [4, 5], Rayleigh-Bénard convection patterns [6–8], turbulence in active matter [9], extreme events in lasers [10], and rogue waves in dissipative optical systems [11]. One question that has attracted considerable attention is how this complexity can be controlled and how these instabilities can be suppressed [12–14]. This is a particularly relevant question in the context of cardiac dynamics. Cardiac tissue is a prototypical example of an excitable medium, where cardiomyocytes are interconnected via gap junctions [15]. Under normal conditions, a planar electrical activation front sweeps through the tissue, but under abnormal circumstances this planar wave can become unstable and the break-up of the front can result in the formation of spiral waves. This disorganized behavior leads to serious heart rhythm disorders, including tachycardia and fibrillation [15–17]. Spiral wave self-organization, however, is not limited to cardiac tissue and is a common feature of excitable media, observed in the brain cortex [18, 19], multicellular aggregates [20], bacterial populations [21], and cytoskeletal filaments [22].

In cardiac tissue slices, the simplest form of a spiral wave rotates around a circular core. Changing the excitability properties of the tissue can destabilize this rigidly rotating spiral wave, resulting in a meandering spiral [23–25] or in wave break-up, leading to spiral defect chaos [26, 27]. Cardiac tissue, has finite thickness and 3D effects may play an important role in the formation of instabilities. The 3D equivalent of a spiral wave is a scroll wave: a stack of 2D spiral waves whose tips form a filament [28, 29]. In sufficiently thick geometries, filaments add degrees of freedom and their dynamics is richer in both homogeneous [29–33], and heterogeneous tissues [34–36]. Conversely, in thin, scroll waves exhibit dynamics similar to the spiral waves in 2D [37].

Successful control of scroll wave instabilities in cardiac tissue has obvious clinical significance and can prevent or even revert tachycardia and fibrillation to normal sinus rhythm [38, 39]. Yet a standard, minimally invasive technique remains debated. Here we demonstrate that introducing boundary layer heterogeneities in a thin computational tissue slab can increase scroll wave stability. Specifically, we show that decreasing the coupling strength between cardiac cells or their excitability properties near the slab boundaries can stabilize scroll waves in the bulk against meandering and break-up instabilities, and even terminate wave activity. Moreover, we theoretically establish a boundary-driven delayed-feedback mechanism underlying the stabilization.

The choice behind boundary layer heterogeneities is motivated by the fact that arrhythmias are often treated by ablation during which tissue is locally destroyed by applying heat, cold, or pulsed electric fields. However, ablation often leads to incomplete lesions, and only renders tissue non-conductive or non-excitable up to a certain depth [40–43], thus creating a significant transmural heterogeneity in which a region with impaired conduction (boundary layer) is sandwiched between conductive (bulk) and non-conductive tissue. Ablation lesions are typically asymmetric in the transmural direction, but can also be symmetric (Fig. 1A). We concentrate on the latter, as the former can be inferred by symmetry arguments (see Supplemental Material (SM) [44]). Furthermore, following previous numerical studies [45–47], we only consider short-term post-ablation effects and neglect possible long-term remodeling and recovery of tissue [48].

We consider an electrically insulated tissue slab of thickness H≪L, where L=5.08 cm is the width of the slab. The slab is divided into a boundary layer of size l and a bulk region of size h/2 along the transmural direction z (Fig. 1B). We model the boundary layer as a region where the coupling strength between cardiac cells is smaller than in the bulk by a factor α. Modulating the coupling strength, rather than excitability, provides a more model-independent approach. Nevertheless, as we detail in the SM [44], altering excitability properties leads to qualitatively similar results. We discretize the z-direction in slices with a thickness corresponding to the size of a cardiomyocyte: $d_{z}=25\;\mu \text{m}$ [49].

To model the excitable dynamics of cardiac cells, we employ the following semi-discrete equation;

(1) $$\partial_{t}u=I_{k}D_{o}\nabla_{⊥}^{2}u+\frac{D_{z}}{d_{z}^{2}}C_{z}-I_{ion},\;k=\left\{0,..,H/d_{z}\right\},$$

where u represents the transmembrane voltage in each cell. The first term describes the coupling in the x−y plane, modeled by isotropic diffusion with a coefficient $D_{o}$, and $I_{k}$ encodes the heterogeneous coupling strength distribution in the different k-slices. The second term describes the discrete coupling along z with a coupling strength $D_{z}/d_{z}^{2}$. Both $I_{k}$ and $C_{z}$ depend on α at the boundary layers (SM [44], Section I). The last term represents the ion currents and is taken from the Fenton-Karma model [50] (parameters in SM [44], Section I).

We first investigate how boundary layer heterogeneities can stabilize the meandering instability in the geometry shown in Fig. 1. For this, we choose parameter values for which in the homogeneous case (α=1) the model Eq. (1) exhibits a scroll wave that rigidly rotates with a frequency $\omega_{o}$ exhibiting a straight filament [51]. Decreasing $\tau_{d}$, a parameter that controls the excitability, the scroll wave starts to rotate faster and eventually undergoes a meandering instability at a critical value $\tau_{d}^{c}=0.384$ ms [52]. As a result, the filament repeats a star-shaped trajectory every 0.5 s as evident from the filament trajectories at the top and bottom slices of the slab (Fig. 1C).

Introducing a boundary layer heterogeneity of size $l=d_{z}$ and strength α=0.004 reduces $\tau_{d}^{c}$, increasing the stability range of rigidly rotating scroll waves (Fig. 1D and Video S1). We note that the heterogeneity need not encompass the entire boundary slice to suppress meandering and can also stabilize scroll waves in the presence of rotational transmural anisotropy and in thin curved geometries (Fig. S1 and Section II-IV in SM [44]).

To systematically explore the stabilization of scroll waves in the presence of boundary layer heterogeneities of size $d_{z}$, we start with a rigidly rotating scroll wave at $\tau_{d}^{o}=0.390$ ms. We then decrease this parameter every 15 rotations in steps of 0.001 ms until we trigger the meandering instability. We first carry out this parameter sweep in the absence of a heterogeneity, which shows that the frequency ω increases linearly as $\tau_{d}$ decreases (open symbols and red line, Fig. 2A). Repeating this procedure in the presence of the heterogeneity reveals a slowing down of the scroll wave and a shift of the meandering bifurcation, $\tau_{d}^{c*}=0.373$ ms (closed symbols in Fig. 2A). Note, however, that the linear dependence between ω and $\tau_{d}$ is almost conserved in the heterogeneous case, but with a slight deviation within the range $\left[\tau_{d}^{c*},\tau_{d}^{c}\right]$, as indicated by the blue dashed line in Fig. 2A.

Next, to elucidate the role of the slab thickness and the strength of the coupling at the boundaries in scroll wave stabilization, we systematically vary H and α and determine $\tau_{d}^{c*}$ and the angular frequency at $\tau_{d}^{o}$. The results can be summarized in two phase diagrams. The first one shows the increase in stability $\text{Δ}\tau_{d}^{c}/\tau_{d}^{c}$, where $\text{Δ}\tau_{d}^{c}=\tau_{d}^{c}-\tau_{d}^{c*}$ (Fig. 2B). The second one displays the frequency ratio $\omega /\omega_{o}$ at $\tau_{d}^{o}$ (Fig. 2C), which is a signature of the boundary layer effect on the scroll wave motion. For a fixed H, both quantities exhibit a non-monotonic behavior, as expected from the equivalence between α=0 and α=1 in our model.

The phase diagrams illustrate that an enhancement of stability is correlated with a reduction in the rotational frequency of the waves. This enhancement becomes larger for thinner slabs, which can be expected since the boundary layer becomes proportionally bigger. Additional computational simulations show that larger boundary layers $\left(l>d_{z}\right)$ increase bulk stabilization for almost all α values (Fig. S3 in SM [44]). This shows that to observe a significant $\text{Δ}\tau_{d}^{c}/\tau_{d}^{c}$, and the corresponding decrease in ω, it is not necessary to reduce the coupling strength by a factor of 100–500. Instead, a reduction that is almost two orders of magnitude smaller can be sufficient if the boundary layer size is large enough. At the same time, larger boundary layers allow a substantial $\text{Δ}\tau_{d}^{c}/\tau_{d}^{c}$ in thicker slabs (Fig. S3B in SM [44]), demonstrating that the stabilization mechanism is robustly present across slab thicknesses with a suitable chosen l [54]. Note that the ratio 2l/H measures the amount of tissue with reduced conductivity, and its impact on the reduction of ω is similar to what has been observed in 2D reaction-diffusion models with randomly distributed weakly conductive inclusions [55]. A further consequence of this ratio is that the degree of stabilization is conserved when considering only one heterogeneous boundary in a slab of thickness H/2 (Fig. S4 in SM [44]).

A relevant division in the phase diagrams occurs at $\alpha_{f}=0.002$ (dashed line in Figs. 2B-C). For $\alpha <\alpha_{f}$, wave propagation from the bulk fails to excite the boundaries, while for $\alpha \geq \alpha_{f}$, it succeeds. In the gray areas in Figs. 2B-C, the introduction of the boundary layers terminates wave activity in the bulk and renders the tissue unexcitable. This behavior is also observed when the boundary layers are thicker (Fig. S3 in SM [44]).

To gain insight into the suppression of the meandering instability, we first analyze separately the cases $\alpha <\alpha_{f}$ and $\alpha \geq \alpha_{f}$. In the former case, ω varies linearly as a function of α for all values of H (Fig. 3A). Furthermore, in this case, the maximum of the potential in the boundary slice, $u_{0}$, increases linearly with α, until $\alpha =5\times 10^{-4}$ (Fig. 3B), but is significantly lower that the maximum value in the bulk slice next to the boundary, $u_{1}\;(\approx 0.92)$. We therefore call this the *leakage* regime: wave propagation in the bulk is not enough to fully excite the boundary slices and the potential in the bulk leaks into the boundary layers (Fig. 3C). The propagation failure of the action potential into the boundary layers resembles wave pinning in discrete systems governed by excitable or bistable dynamics [56–60], and the growth of $\max\left(u_{0}\right)$ can be interpreted as a precursor to wave unpinning.

The linear decrease of ω, as a function of α, can be analytically addressed in the case $\alpha <\alpha_{f}$. For this, we consider a tissue slab of size $H=2d_{z}$, i.e., one bulk slice sandwiched by two boundary layers. Since $u_{0}\ll u_{1}$, the dynamics can be analyzed by considering only the bulk slice, where $C_{z}=-2\alpha D_{z}/d_{z}^{2}u_{1}+O\left(\alpha u_{0}\right)$. This negative linear term implies a reduction of excitability in the bulk. Similar to previous works [33, 61], we perform a weakly nonlinear analysis and find that the O(α) correction in ω is $\text{Δ}\omega \propto -2D_{z}\alpha /d_{z}^{2}$ (SM [44], Section VII). Consistent with our numerical results, this analysis shows that the decrease of excitability in the bulk, governed by the boundary layer heterogeneities, reduces ω. Although this analysis is only valid for a non-meandering spiral wave in the homogeneous case, i.e. $\tau_{d}>\tau_{d}^{c}$, our numerical results support the idea that a reduction in ω plays a role in the delay of the meandering instability (Figs. 2B-C).

When $\alpha \geq \alpha_{f}$, an abrupt unpinning-like transition lets bulk waves fully excite the boundary layers (SM [44], Sec. VIII), switching to a feedback regime where the boundary regulates the bulk and a bulk scroll drives a boundary spiral, producing a nontrivial voltage difference at the bulk-boundary layer interface (Fig. 3D). We note that the boundary layers usually cannot excite the bulk on their own. This type of nonreciprocal propagation failure has been studied in 1D and 2D reaction-diffusion models [62–65], where it was shown that sharp jumps in conductivity are the main cause of this behavior. In our case, however, this is not the whole story as the ratio 2l/H controls the ability of the boundaries to excite the bulk. When 2l/H≲0.5, the boundaries fail to excite the bulk even as α→1, and when 2l/H≳0.5, the boundaries can activate the whole slab in the feedback regime.

When $l=d_{z}$, the problem can be simplified by deriving a 2-slice model, consisting of only a boundary layer slice and a bulk slice. For this, we use the fact that for all values of α and h, our simulations show that in the bulk the term $C_{z}=D_{z}\partial_{zz}u$ is approximately independent of z [66]. Then, in the rotational frame of reference, we use the symmetry of our geometrical set-up and solve the Laplace equation in one half of the slab with the Dirichlet boundary condition $u=u_{m}$ at z=0, where $u_{m}=u_{H/2dz}$ is the transmembrane voltage at the middle slice, and the Neumann boundary condition $\partial_{z}u=\alpha \left(u_{0}-u_{1}\right)/d_{z}$ at z=h/2. The solution, back in discrete form and for a general size l, is $u_{k}=u_{m}+\chi_{k}\left(u_{b}-u_{m}\right)$, where $\chi_{k}=4\alpha {\left[kd_{z}-l-h/2\right]}^{2}/\left(4hd_{z}+\alpha h^{2}\right)$ with k running from $k=l/d_{z}$ to $k=(H-l)/d_{z}$, and $u_{b}=u_{l/d_{z}-1}$ is the boundary transmembrane voltage next to the bulk. The $u_{k}$ fields can be introduced in Eq. (1), reducing the full quasi-2D equations into a far simpler 2-slice model

(2) $$\begin{array}{l} \partial_{t}u_{m}=D_{o}\nabla_{⊥}^{2}u_{m}-I_{ion}^{m}+2\frac{D_{z}}{d_{z}^{2}}\chi_{m+1}\left(u_{b}-u_{m}\right)\\ \;\partial_{t}u_{b}=\alpha D_{o}\nabla_{⊥}^{2}u_{b}-I_{ion}^{b}+\alpha \frac{D_{z}}{d_{z}^{2}}\left(1-\chi_{b+1}\right)\left(u_{m}-u_{b}\right), \end{array}$$

where $I_{ion}^{m}$ and $I_{ion}^{b}$ are the ion currents at the middle slice and at the boundaries next to the bulk, respectively. We numerically integrate Eq. (2) using as initial condition a rigidly rotating spiral for $u_{m}$ and a non-excited state for $u_{b}$. The results are in perfect agreement with the full model Eq. (1) in terms of ω, $\text{Δ}\tau_{d}^{c}$ (Fig. 4A), and even core size of the scroll wave at the middle slice $R_{m}$ (Section IX and Fig. S6 in the SM [44]). For $l>d_{z}$, it is still possible to reduce the bulk into a single slice, but this strategy cannot be extended to the boundary layer. However, one can approximate the correction in the 2-slice model for larger boundary layers (Section X in the SM [44]), and find again an excellent agreement for $l=2d_{z}$ (Fig. 4B).

In the feedback regime, the bulk, represented by $u_{m}$, experiences not only a decrease in excitability caused by $-2D_{z}\chi_{m+1}u_{m}/d_{z}^{2}$, but also a continuous forcing from the boundaries given by $2D_{z}\chi_{m+1}u_{b}/d_{z}^{2}$. To shed light on the nature of this forcing, close to $\alpha_{f}$, we can simplify the equation of $u_{b}$ in (2) by assuming that its dynamics is mainly driven by $u_{m}$; $\partial_{t}u_{b}\approx \alpha D_{z}\left(1-\chi_{b+1}\right)\left(u_{m}-u_{b}\right)/d_{z}^{2}$. This expression can be integrated to find $u_{b}$, which, when introduced into the first equation of (2), gives

(3) $$\partial_{t}u_{m}\approx D_{o}\nabla_{⊥}^{2}u_{m}-I_{\text{ion}}^{m}-\frac{1}{T_{b}}u_{m}+\frac{1}{T_{b}T_{bl}}\int_{0}^{t}e^{-\frac{t-t^{\prime }}{T_{bl}}}u_{m}^{\prime }dt^{\prime },$$

where $T_{b}^{-1}=2D_{z}\chi_{m+1}/d_{z}^{2}$, $T_{bl}^{-1}=\alpha D_{z}\left(1-\chi_{b+1}\right)/d_{z}^{2}$ and $u_{m}^{\prime }=u_{m}\left(x,y,t^{\prime }\right)$. Integration of this forced model reproduces reasonably well the enhancement of stability (Fig. 4A). Thus, this simplification reveals that wave stabilization is the consequence of a reduction in bulk excitability and a boundary-driven delayed-feedback. A useful lesson of Eq. (3) is that to sustain wave dynamics the feedback from the boundaries needs to be fast enough to balance the loss in bulk excitability, i.e., $T_{b}/T_{bl}=h/2d_{z}>1$. This last condition is not satisfied for the case $h=2d_{z}$, where we observe wave termination when $l=d_{z}$ (Fig. 2B), indicating that the bulk cannot support waves due to a loss in excitability. The forced model also captures $\text{Δ}\tau_{d}^{c}$ when $l=2d_{z}$ (Fig. 4B), and reveals that larger boundary layers correlate with smaller $T_{b}/T_{bl}$ (Section X and Fig. S7 in the SM [44]).

The stabilizing properties of boundary layer heterogeneities are not limited to the meandering instability but can be extended to spiral wave break-up. To illustrate this, we modify the electrophysiological parameters (SM [44]; Section I) in the FK model such that a rigidly rotating spiral wave in homogeneous conditions undergoes break-up driven by discordant alternans [26]. We use the computationally efficient Eq. (2) to implement the boundary layer effects, but we have verified that the full quasi-2D equations give similar results. The simulations reveal that the introduction of boundary layers can enhance the stability of scroll waves and prevents break-up initiation (Section XI and Fig. S8 in the SM [44]).

In summary, we prove that boundary layer heterogeneities can control scroll wave dynamics through either heterogeneities in the coupling strength or the tissue excitability (SM [44], Section XII). Unlike prior studies that emphasize destabilizing gradients along the filament orientation [67–69], our contribution focuses on the stabilizing role and reveals theoretically the stabilization mechanism. It results from a balance between reduction in bulk excitability and a boundary-driven delayed-feedback: the boundary serves as a slowing zone that receives the incoming bulk wave and feeds it back with a delay that depends on geometry $\left(d_{z},h,l\right)$ and coupling strength $\left(D_{z},\alpha D_{z}\right)$. While feedback-mediated stabilization of spiral waves has been studied before using imposed measurements and controllers [70], in our system the feedback emerges intrinsically from a bulk-boundary layer interaction. Additionally, we identify a leakage-feedback transition that resembles a pinning-unpinning transition. This underscores how the semidiscrete limit, physiologically plausible in thin heterogeneous cardiac tissue, uncovers novel dynamical regimes in excitable media.

Our work could pave the way for new control strategies in cardiac research, where the tuning of boundary heterogeneities could enable bulk waves to self-regulate themselves. Of course, it will be necessary to characterize tissue properties to avoid scenarios in which the partially ablated region could favor wave break-up of incoming planar waves, a common ablation caveat [71, 72]. Cardiac optogenetics [73–77] may provide a way to precisely manipulate boundary properties and experimentally test our mechanism. Future work could investigate the long-term effects of ablation, where healing processes and nonlocal mechanical effects may play a relevant role. Finally, we expect boundary-driven control to be applicable to other excitable systems, such as Rho GTPase wave activity in the actin cortex of eukaryotic cells [78] and the Belousov-Zhabotinsky chemical reaction [4], motivating further experiments.

## Supplementary Material

Supplement 1

## Figures and Tables

**FIG. 1.**
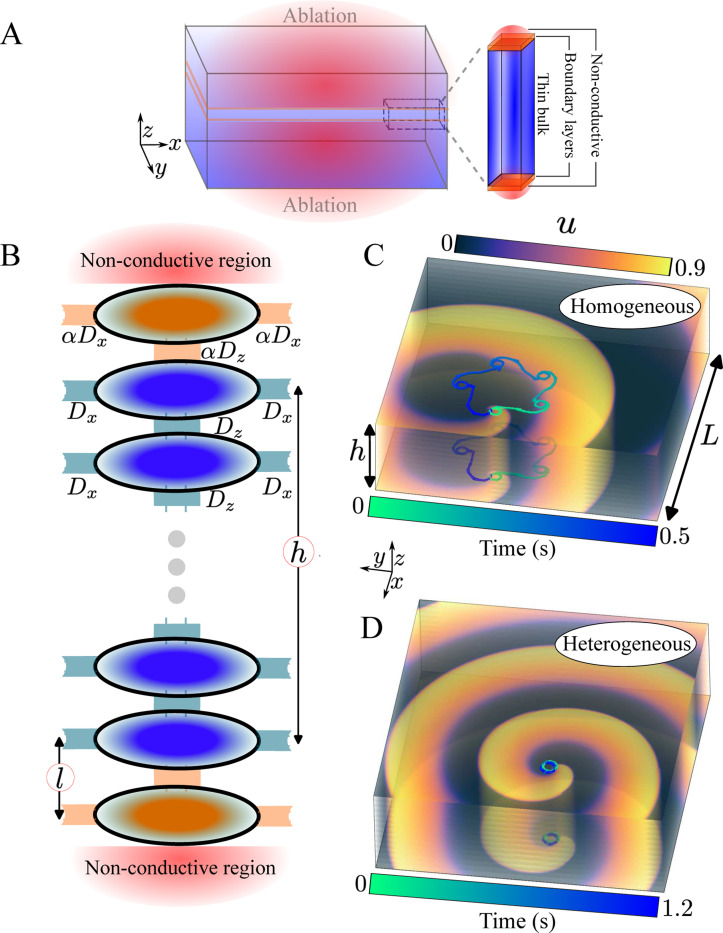
Boundary layer effects. (A) Cartoon of a non-transmural ablation. (B) Schematic representation of the discrete coupling between cardiomyocytes along the transmural direction. (C) Star-like tip trajectories and the variable u in the top and bottom slice for the homogeneous case at $\tau_{d}=0.382$ ms, $H=14d_{z}$. (D) As in B with boundary layer heterogeneities of strength α=0.004 and size $d_{z}$.

**FIG. 2.**
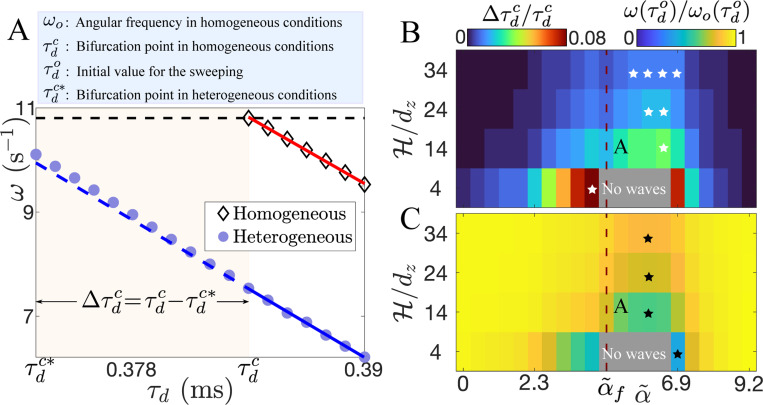
Control of meandering instability with $l=d_{z}$. (A) ω vs $\tau_{d}$ in homogeneous and heterogeneous conditions. The horizontal black dashed line emphasizes $\omega_{o}\left(\tau_{d}^{c}\right)$. The solid lines indicate linear fits. (B-C) Phase diagrams in $\tilde{\alpha }-H/d_{z}$ space showing $\text{Δ}\tau_{d}^{c}/\tau_{d}^{c}$ and $\omega /\omega_{o}$ at $\tau_{d}^{o}$, respectively. Here $\tilde{\alpha }=log(\alpha )+c$ with c=10.82 [53]. The white (black) stars indicate $\max\left(\text{Δ}\tau_{d}^{c}/\tau_{d}^{c}\right)\;\left(\min\left(\omega /\omega_{o}\right)\right)$ for each H.

**FIG. 3.**
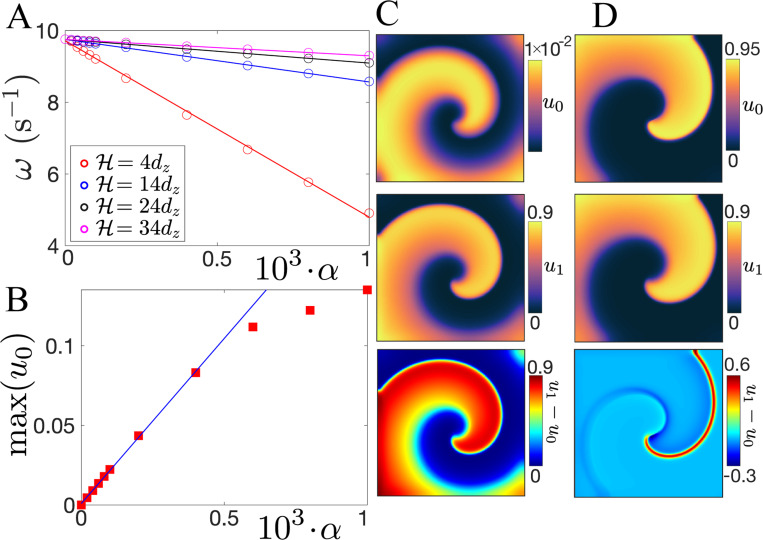
Dynamical regimes of scroll waves. (A) ω vs α for different H in the leakage regime. (B) $\max\left(u_{0}\right)$ vs α ($H=14d_{z}$ and $l=d_{z}$). (C-D) $u_{o}$, $u_{1}$ and $u_{1}-u_{o}$ in the (C) leakage $\left(\alpha =4\times 10^{-5}\right)$ and (D) feedback $\left(\alpha =4\times 10^{-3}\right)$ regimes.

**FIG. 4.**
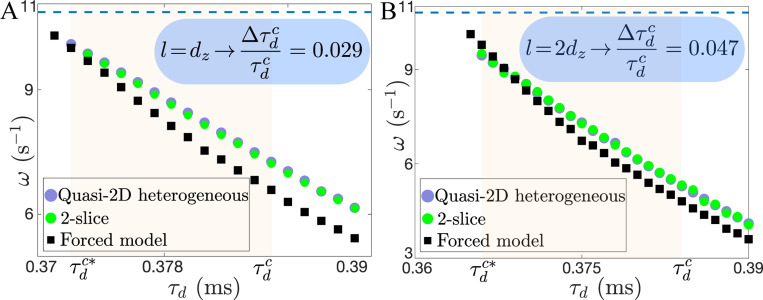
ω vs $\tau_{d}$ in the different models for the case $H=14d_{z}$ and α=0.005. (A) $l=d_{z}$, (B) $l=2d_{z}$. The dashed line corresponds to the $\omega_{o}\left(\tau_{d}^{c}\right)$, and the shaded area to $\text{Δ}\tau_{d}^{c}$.
