# Supplementary material for: Boundary-driven delayed-feedback control of spatiotemporal dynamics in excitable media
Source: ArXiv. 2025 Dec 1:arXiv:2409.00183v3. Preprint. [Version 3] (PMC12687857)
Supplement: Supplement 1 [file NIHPP2409.00183v3-supplement-1.pdf]

Supplemental Material for Boundary-driven delayed-feedback control of spatiotemporal dynamics in excitable media

Sebastián Echeverría-Alar and Wouter-Jan Rappel
Department of Physics, University of California, San Diego, California, 92093, USA
(Dated: December 2, 2025)

Contents

I. Electrophysiological model 2

II. Scroll wave stabilization in the presence of a localized transmural heterogeneity 2

III. Transmural rotational anisotropy 3

IV. Boundary layers in thin curved geometries 3

V. Larger boundary layers and wave termination 4

VI. Asymmetric heterogeneity distribution 4

VII. Weakly nonlinear analysis in the leakage regime $\alpha < \alpha_f$ 5

VIII. Pinning-unpinning-like transition at $\alpha = \alpha_f$ 7

IX. Core radius in the multiple models 7

X. Extension of the reduced models to larger boundary layers 7

XI. Control of spiral wave break-up through boundary layer heterogeneities 8

XII. Reduced excitability of boundary layer cells 9

XIII. Discretization and propagation failure on the $x-y$ plane 10

XIV. Numerical implementation of the quasi-2D model in WebGL 10

XV. Video caption 11

I. Electrophysiological model

The reaction-diffusion model chosen to explore the effects of boundary layer heterogeneities in the stability of scroll waves, is a semi-discrete version of the well-established Fenton-Karma (FK) model [1]. In this model, the evolution of the transmembrane action potential u obeys

$$\partial_t u = \mathcal{I}_k D_o \nabla_\perp^2 u + \frac{D_z}{d_z^2} C_z - \frac{I_{ion}}{C_m}, \tag{1}$$

where $D_o = 0.001$ cm^2/ms is a diffusion coefficient and $\mathcal{I}_k$ is a function controlling the heterogeneity distribution in each slice:

$$\mathcal{I}_k = \begin{cases} \alpha, & 0 \leq kd_z < l \cup \mathcal{H} - l < kd_z \leq \mathcal{H} \\ 1, & l \leq kd_z \leq \mathcal{H} - l \end{cases}. \tag{2}$$

Here, the subscript $k = \{0, .., \mathcal{H}/d_z\}$ labels the tissue slice along the transmural direction. The second term, C_z, describes discrete coupling along the z direction and is given by

$$\mathcal{C}_z = \Big[\mathcal{Q}_{k+1}(u_{k+1} - u_k) + \mathcal{Q}_{k-1}(u_{k-1} - u_k)\Big], \tag{3}$$

where $\mathcal{Q}_{k+1} = min(\mathcal{I}_k, \mathcal{I}_{k+1})$ and $\mathcal{Q}_{k-1} = min(\mathcal{I}_k, \mathcal{I}_{k-1})$. Consistent with experimental data, we use a transverse diffusion constant $D_z = D_o/5$. C_m is the membrane capacitance and $I_{ion} = I_{fi} + I_{so} + I_{si}$ is the sum of ionic currents. The membrane capacitance is dimensionless and equal to 1 and has thus been omitted in the main text. The currents depend on electrophysiological parameters and describe the following excitable dynamics

$$I_{fi}(u, v) = -\frac{v}{\tau_d}\Theta(u - u_c)(1 - u)(u - u_c), \tag{4}$$

$$I_{so}(u) = \frac{u}{\tau_o}\Theta(u_c - u) + \frac{1}{\tau_r}\Theta(u - u_c), \tag{5}$$

$$I_{si}(u, w) = -\frac{w}{\tau_{si}}(1 + \tanh(k(u - u_{c,si}))). \tag{6}$$

The gating variables v and w evolve according to local dynamics:

$$\partial_t v = \frac{1}{\tau_v^-(u)}\Theta(u_c - u)(1 - v) - \frac{1}{\tau_v^+}\Theta(u - u_c)v, \tag{7}$$

$$\partial_t w = \frac{1}{\tau_w^-}\Theta(u_c - u)(1 - w) - \frac{1}{\tau_w^+}\Theta(u - u_c)w, \tag{8}$$

where $\tau_v^-(u) = \Theta(u - u_v)\tau_{v1}^- + \Theta(u_v - u)\tau_{v2}^-$. In our simulations, all electrophysiological parameters are kept constant (and listed in Table S1), except τ_d, which controls the excitability in Eq. (1), and is used as the bifurcation parameter to trigger either a meandering or a break-up instability. The system of equations is numerically integrated on a slab of size $L \times L \times \mathcal{H}$ (see Fig.1 in the main text). In each $x - y$ slice, the Laplacian is approximated by a 5-point stencil with a spatial discretization size $\Delta x = 0.0254$ cm, and in the transmural (z) direction, we used the discretization reported in the main text. We have verified that the effects of choosing smaller values of Δx are negligible (see Section XIII). The whole slab is bounded by non-flux boundary conditions. We used a forward Euler method for temporal integration with a time step $\Delta t = 0.01$ ms for the quasi-2D simulations, $\Delta t = 0.08$ ms for the reduced 2D simulations, and $\Delta t = 0.005$ ms for the simulations in Section XIII. The numerical implementation was done in Matlab and WebGL [2]. The latter is briefly detailed in Section XIV.

II. Scroll wave stabilization in the presence of a localized transmural heterogeneity

In the main text, we focus on the simple scenario in which the heterogeneity distributions are uniform in the $x - y$ directions and encompass the whole slice. Here, we ask ourselves if it is possible to stabilize the meandering instability with a localized heterogeneity in the $x - y$ plane and if this heterogeneity can trap a meandering scroll wave. To answer this question, we introduce a circular heterogeneity in the upper and lower slice of a $2L \times 2L \times \mathcal{H}$ slab. The diameter of the heterogeneity is taken as $L_{het} = 4$ cm and the reduction of conduction is parameterized by $\alpha = 0.004$. Note that this reduction now occurs not only in the transmural direction but also in the $x - y$ plane. Fig. S1 illustrates snapshots of a spiral wave that is meandering in the absence of a boundary layer heterogeneity ($\tau_d = 0.382$). The introduction of the circular heterogeneity, however, stabilizes and traps the meandering wave: once the filament enters the heterogeneous region (circular shaded region), it starts to exhibit locally a rigid rotation behavior (t_3) and after a transient of around $18T_{rot}$, the final equilibrium state is a rigidly rotating scroll wave (t_4).

Table S1: Electrophysiological and numerical parameters used for all numerical simulations in this study. The first (second) row corresponds to the set of parameters for which a spiral wave, at a critical τ_d value, undergoes a meandering (break-up) instability in homogeneous conditions.

Instability	C_m (-)	u_c (-)	τ_o (ms)	τ_r (ms)	τ_{si} (ms)	k (-)	$u_{c,si}$ (-)	u_v (-)	τ_{v1}^- (ms)	τ_{v2}^- (ms)	τ_v^+ (ms)	τ_w^- (ms)	τ_w^+ (ms)	L (cm)	Δx (mm)	d_z (μm)
Meandering	1	0.13	9	33	29	15	0.5	0.04	9	8	3.3	60	250	5.08	0.254	25
Break-up	1	0.15	9	34	26.5	15	0.45	0.04	15.6	5	3.3	80	350	12.24	0.254	25

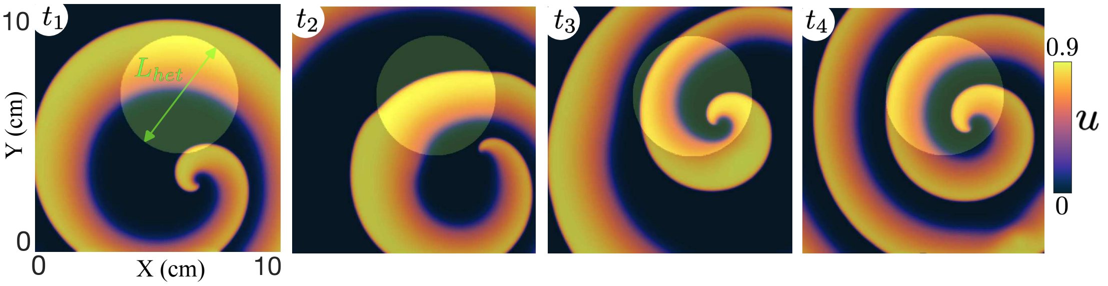

Figure S1: Scroll wave interacting with a localized boundary layer heterogeneity in the FK model. The temporal sequence $t_1 = 0 < t_2 < t_3 < t_4 = 19T_{rot}$, with $T_{rot} = 122$ ms, describes the trapping of a scroll wave observed at the middle of a tissue slice with thickness $\mathcal{H} = 14d_z$. The boundary layers have a volume $\pi L_{het}^2 l/4$ and in this particular simulation $l = d_z$ and $L_{het} = 4$ cm.

III. Transmural rotational anisotropy

Cardiac tissue is inherently anisotropic [3]. The elongated cardiomyocytes transport ions more efficiently along their longitudinal axis than along their transverse axis. In 2D simulations, this anisotropy is usually not an issue, as differences in conductivity along the x and y directions can be rescaled, as long as the orientation of the cardiac cells does not vary in space. In 3D, however, the situation becomes more subtle: even if the orientation of the cells is independent of x and y, it can change along the z-axis. Motivated by images of the myocardial architecture in the ventricles, the resulting transmural dependence is generally modeled in simulations as a constant change in cardiomyocytes orientation (rotational anisotropy) from endocardium to epicardium [1]. Simulations that incorporated rotational anisotropy have revealed that it can only de-stabilize a spiral wave filament if the tissue thickness is sufficiently large [1, 4]. In other words, in thin geometries such as the atrial walls, rotational anisotropy will only have a negligible effect in scroll wave dynamics.

We have verified that in our thickest tissue slab ($\mathcal{H} = 34d_z$), even using a strong rotational rate of 30°/mm [1, 4], transmural rotational anisotropy does not modify the critical τ_d values reported in the main text, neither in the homogeneous case (τ_d^c) nor in the heterogeneous (τ_d^{c*}) case with $l = d_z$ and $\alpha = 0.004$. Thus, it is reasonable to neglect transmural anisotropy.

IV. Boundary layers in thin curved geometries

To show the stabilization of scroll waves against meandering instabilities in more complex geometries, we consider a half-ellipsoidal shape with a nearly constant thickness $\mathcal{H}$ (a cross-sectional cut is shown in Fig. S2A). This geometrical representation has been used before to study scroll wave dynamics based on morphological measurements of the human heart [5]. The introduction of the boundary layers can be motivated by two possible incomplete ablation lesions. For example, if the ablation produces a gap, of size h, such that the curvature from the heart surfaces can be neglected compared to the spatial dimension of the gap, as shown in Fig. S2B, our theoretical framework remains valid given that L_{het} is bigger than the spiral core (see Fig. S1). A less trivial case is presented in Fig. S2C, where, possibly due to multiple unfinished ablations with different orientations, the gap displays a distinct curvature.

We use the phase field approach to model the case depicted in Fig. S2C, where non-flux boundary conditions on the inner and outer surfaces of the thin half-ellipsoidal geometry are enforced via a scalar field φ [6]. This quantity, defined on a Cartesian grid, is equal to 1 inside the half-ellipsoid and is 0 outside. The interface connecting both values is generated through a diffusion process, using $\phi = 1$ in the region of interest and 0 elsewhere as initial condition. By varying the total evolution time of the diffusive dynamics, one can control the smoothness (or sharpness) of the interface. We implement the phase field so that any one-dimensional cut in the transmural direction closely matches $\mathcal{I}_k$ (see Eq. 2 in the main text) with $\mathcal{H} = 14d_z$ and $l = d_z$. For the homogeneous scenario, i.e., without a boundary layer, we apply the Closest Point Method, which allows to impose non-flux boundary conditions on curved surfaces (see [7] for details). Notice that, because our geometries are thin, the quasi-2D homogeneous scenario is equivalent to

the 2D one. Once the geometries are generated, we integrate the FK model using the parameters for which the spiral wave undergoes a meandering instability (first row of Table S1).

The initial condition is a scroll wave placed on top of a half-ellipsoid. The epicardial transmembrane voltage is shown in Fig. S2D for a major axis $a = 4$ cm and a minor axis $b = 2$ cm. Then, as in the main text, we decrease τ_d from $\tau_d^o = 0.390$ until a meandering instability is triggered. In the homogeneous case, the critical value of τ_d, τ_d^c, is increased compared to the non-curved geometry (cyan symbols vs. the blue dashed line in Fig. S2E). In the presence of boundary layers the meandering stability is enhanced, $\tau_d^{c*} < \tau_d^c$, (magenta symbols, Fig. S2E) with a slight increase in τ_d^{c*} compared to the non-curved case (red dashed line). This enhancement is robust against variations of the curvature, controlled by the ratio a/b.

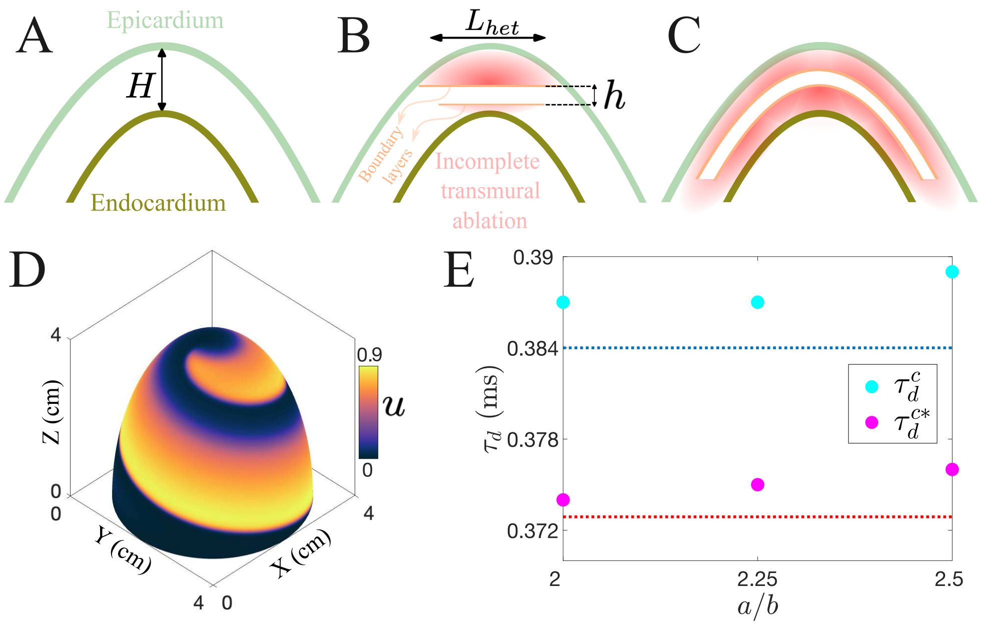

Figure S2: Boundary layer effect on the meandering instability in a thin half-ellipsoidal geometry. (A) Two-dimensional cross-sectional cut of the curved geometry. (B-C) Schematic representations of incomplete transmural ablation lesions, represented in red, in the curved geometry. In B, the lesion is small compared to the inverse of the curvature and the system can be treated as in the main text. In C, the size of the lesion cannot be neglected compared to the curved geometry. (D) Initial transmembrane voltage at the outer (epicardial) surface of the half-ellipsoid. (E) Critical τ_d values for the homogeneous (τ_d^c) and the heterogeneous cases (τ_d^{c*}) with $\alpha = 0.004$. The dashed lines correspond to the critical values in the non-curved geometry (see main text).

V. Larger boundary layers and wave termination

In the main text, we focus on the scroll wave stabilization using boundary layer heterogeneities of size $l = d_z$. However, we have verified that the delay of the meandering instability by slowing down scroll waves is also present when $l > d_z$. Fig. S3 shows the change in the enhancement of stability of scroll waves, $\Delta\tau_d^c/\tau_d^c$, in tissue slab of thicknesses $\mathcal{H} = 14d_z$ (A) and $\mathcal{H} = 24d_z$ (B) in the presence of boundary layers of sizes $l < \mathcal{H}/4$. This choice implies that we only consider situations where the bulk is bigger than the boundary layers; $h > 2l$. When $l > 2d_z$, our simulations show that the leakage-transition feedback is shifted towards higher values of α, as indicated by the dashed line in Fig. S3.

In the feedback regime ($\alpha \geq \alpha_f$), the enhancement of stability is larger for thicker boundary layers. This is related to the fact that the ratio $2l/h$ becomes closer to 1. Additionally, we observe that in thicker boundary layers, a significant increase in $\Delta\tau_d^c/\tau_d^c$ can be achieved for higher α values.

As in the main text, the gray zones indicate regions in which wave activity is terminated. For the larger slab thicknesses, termination occurs over a range of boundary layer sizes. Furthermore, this termination also shifts towards larger values of α. Thus, smaller differences in conductivity between the bulk and the boundary layer will be able to terminate all wave activity.

VI. Asymmetric heterogeneity distribution

The symmetry of the heterogeneity distribution used in the main text (see Fig. S4A) allows us to easily extend our results to asymmetric distributions in thin tissue slabs. Fig. S4B displays an asymmetric distribution consisting of a tissue slab of half the size in the symmetric case and with a heterogeneous boundary layer in only one boundary. This

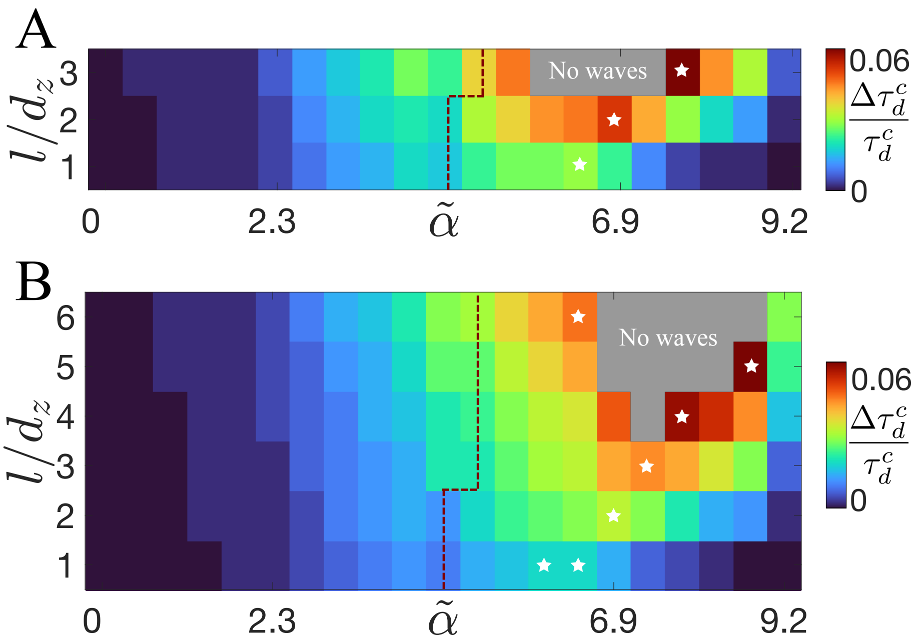

Figure S3: Effects of boundary layer size l in the enhancement of scroll wave stability $\Delta\tau_d^c/\tau_d^c$ for different α values, where $\tilde{\alpha} = log(\alpha) + 10.82$, and for two slab thicknesses: (A) $\mathcal{H} = 14d_z$ and (B) $\mathcal{H} = 24d_z$. The dashed red lines indicate the propagation failure along the transmural direction. Gray regions: termination of all wave activity. The white stars indicate $\max(\Delta\tau_d^c/\tau_d^c)$ for each boundary layer size.

new numerical setup, as in the symmetric case, is bounded by non-flux boundary conditions. Similar to the analysis in the main text, we sweep the excitability parameter τ_d starting from a rigidly rotating scroll wave and found that the angular frequency and the enhancement of stability are equal when comparing the symmetric and asymmetric cases for different values of α and $\mathcal{H}$ (Figs. S4C-E). We notice that in both $\mathcal{H} = 4d_z$ (symmetric) and $\mathcal{H} = 2d_z$ (asymmetric) cases, scroll waves terminate in the presence of a heterogeneity with strength $\alpha = 0.004$.

VII. Weakly nonlinear analysis in the leakage regime $\alpha < \alpha_f$

Due to the symmetry of our problem, one can consider only one half of the tissue slab. For example, in the case of a slab with three slices and with a heterogeneity of size $l = d_z$ and strength α, we can write 6 2D equations

$$\begin{aligned}
\partial_t u_1 &= D_x \nabla_\perp u_1 + 2\alpha \frac{D_z}{d_z^2}(u_0 - u_1) - \frac{I_{ion}}{C_m} \\
\partial_t v_1 &= F(u_1, v_1) \\
\partial_t w_1 &= G(u_1, w_1) \\
\partial_t u_0 &= \alpha D_x \nabla_\perp u_0 + \alpha \frac{D_z}{d_z^2}(u_1 - u_0) - \frac{I_{ion}}{C_m} \\
\partial_t v_o &= F(u_o, v_o) \\
\partial_t w_o &= G(u_o, w_o).
\end{aligned} \tag{9}$$

Here the index 0 refers to the boundary layer and the index 1 refers to the bulk. In the leakage regime, $u_1 \gg u_0$, we only need to take into account equations in the bulk since $u_0 - u_1 \approx -u_1$. In other words, the bulk receives a negligible amount of current from the boundary.

Our analysis relies on the linearization of some of the nonlinear terms in Eq. (9). Therefore, we need to smooth the Heaviside functions and we replace these functions by sigmoidal functions; $\Theta(q) = (1 + \tanh(s_f q))/2$ with $s_f = 15$. This changes the excitability of the system and, therefore, the behavior and shape of spiral waves in the parameter set of Table S1. However, it is still possible to trigger a meandering instability, now at $\tau_d^c = 0.348$ instead of 0.384 as for the Fenton-Karma model considered in the main text.

We numerically solve the bulk part of Eq. (9) with $\alpha = 0$ and smoothed Heavisides in polar coordinates where the initial condition is a spiral wave generated from direct numerical simulations in a cartesian grid, which is interpolated to a polar grid centered at the spiral core center. The polar geometry consist of a disk of radius $R_{disk} = 2.29$ cm with

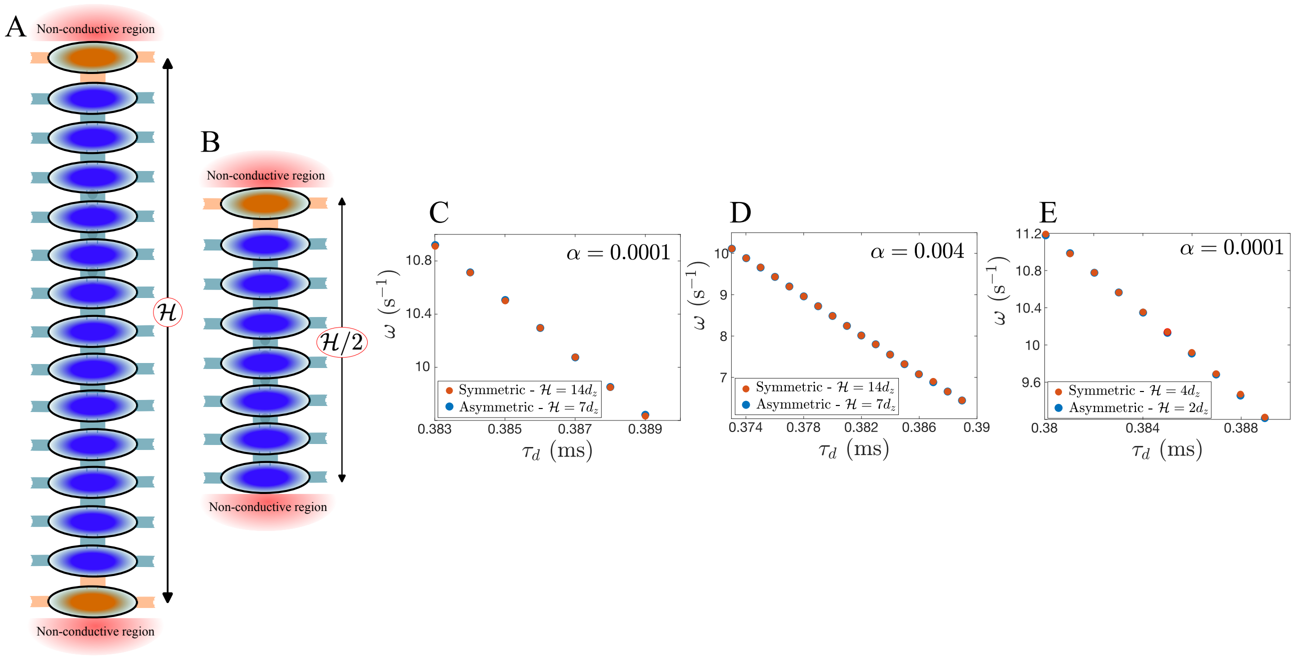

Figure S4: Comparison between symmetric and asymmetric heterogeneity distributions. (A-B) Schematic representation of the coupling along the transmural direction (see Fig. 1 in the main text for further detail). (C-E) Angular frequency as a function of the excitability parameter τ_d for different values of α and $\mathcal{H}$.

a spatial discretization $\Delta r = \Delta x$ in the radial direction and 128 points along the angular (θ) direction. In the radial direction a 4-point stencil is used with non-flux boundary conditions at $r = 0$ and $r = R_{disk}$, and a spectral method is used to discretize the angular terms of the Laplacian [8]. The equations are integrated in time with an explicit Euler method with $\Delta t = 0.0001$ ms. The steady state, achieved after 15 rotations of the spiral wave at $\tau_d = 0.355$, is described by the spiral solution $\{u_1, v_1, w_1\}^{[0]}$ with an angular frequency ω_o.

In the following, we perform a weakly nonlinear analysis around the homogeneous solution in the rotational frame of reference $\partial_t = \omega\partial_\theta$ to characterize how the frequency ω varies as a function of the heterogeneity strength α. Introducing the ansatz $\mathbf{u}_1^{[0]} + \alpha\mathbf{u}_1^{[1]} + ...$, $\omega = \omega_o + \Delta\omega + ...$ in the bulk terms of Eq. (9) and considering that $\Delta\omega \sim \alpha$, we find at $\mathcal{O}(\alpha)$ a linear system of equations for the nonlinear correction; $\mathcal{L}\{u_1, v_1, w_1\}^{[1]} = \mathbf{b}$ where

$$\mathcal{L} = \begin{pmatrix} D_x\nabla_\perp^2 + \omega_o\partial_\theta - \dfrac{\partial\hat{I}_{ion}}{\partial u_1} & \dfrac{\partial\hat{I}_{ion}}{\partial v_1} & \dfrac{\partial\hat{I}_{ion}}{\partial w_1} \\ \dfrac{\partial\hat{F}}{\partial u_1} & \omega_o\partial_\theta + \dfrac{\partial\hat{F}}{\partial v_1} & 0 \\ \dfrac{\partial\hat{G}}{\partial u_1} & 0 & \omega_o\partial_\theta + \dfrac{\partial\hat{G}}{\partial w_1} \end{pmatrix} \tag{10}$$

and

$$\mathbf{b} = -\Delta\omega\partial_\theta \begin{pmatrix} u_1 \\ v_1 \\ w_1 \end{pmatrix}^{[0]} + 2\alpha\frac{D_z}{d_z^2}\begin{pmatrix} u_1 \\ 0 \\ 0 \end{pmatrix}^{[0]}. \tag{11}$$

The terms $\hat{I}_{ion}$, $\hat{F}$ and $\hat{G}$ in Eq. (10) depend on the smoothed version of the Heaviside functions. To solve the linear system, we must introduce an inner product to apply a solvability condition, i.e., the linear equation will have solution if and only if $\mathbf{b}$ is orthogonal to the $Ker\{\mathcal{L}^\dagger\}$. Based on previous studies [9], we consider the inner product

$\langle \mathbf{g}|\mathbf{h}\rangle = \int\int \mathbf{f}\cdot\mathbf{g} r dr d\theta$, and the elements of the kernel $Ker\{\mathcal{L}^\dagger\} = \{\bar{u}_1, \bar{v}_1, \bar{w}_1\}$ are calculated numerically with a precision $\mathcal{O}(10^{-3})$. Finally, we apply the solvability condition $\langle\{\bar{u}_1, \bar{v}_1, \bar{w}_1\}|\mathbf{b}\rangle = 0$ and obtain

$$\Delta\omega = 2\alpha\frac{D_z}{d_z^2}\frac{\langle\{\bar{u}_1, \bar{v}_1, \bar{w}_1\}|\{u_1, 0, 0\}^{[0]}\rangle}{\langle\{\bar{u}_1, \bar{v}_1, \bar{w}_1\}|\partial_\theta\{u_1, v_1, w_1\}^{[0]}\rangle} = 2\alpha\frac{D_z}{d_z^2}\frac{A}{B}, \tag{12}$$

with $A/B = -0.81$.

VIII. Pinning-unpinning-like transition at $\alpha = \alpha_f$

For $\alpha < \alpha_f$, the boundary layer can not be excited by the bulk. As a consequence, the wave is pinned to the boundary-bulk interface. In contrast, when $\alpha \geq \alpha_f$, wave propagation is possible from the bulk to the boundary and the wave is unpinned. This sharp pinning-unpinning-like transition can be visualized by plotting the maximum value of the boundary transmembrane voltage u_0 as a function of α, as shown in Fig. S5.

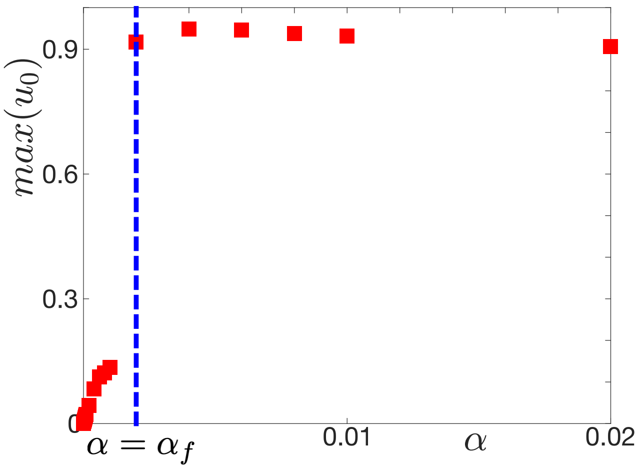

Figure S5: Maximum boundary voltage as a function of α in both leakage and feedback regimes for $\tau_d = 0.390$, $\mathcal{H} = 14d_z$ and $l = d_z$.

IX. Core radius in the multiple models

The core radius R_m of the scroll wave, measured in the middle slice of the thin slab, decreases as the τ_d parameter is also reduced (Fig. S6). This behavior is quantitatively reproduced by the 2-slice model and qualitatively emulated by the forced model. In the simulations of the forced model, Eq. (3) in the main text, the integral is discretized using the trapezoidal rule, and the initial condition is a rigidly rotating spiral.

X. Extension of the reduced models to larger boundary layers

When $l > d_z$, it is still possible to reduce the bulk into a single slice by solving a Laplace equation. However, this technique cannot be applied to simplify the boundary layer, and a reduced model will need to take into account the z-dependence of $\mathcal{C}_z$ in every boundary slice. This will result in a model with $1 + l/d_z$ slices: the reduced bulk slice, the slice at the interface bulk-boundary layer, and the rest of $l/d_z - 1$ slices within the boundary layer. Following the approach of the main text, a 3-slice model for the case $l = 2d_z$ can be written as

$$\begin{aligned}
\partial_t u_m &= D_o\nabla_\perp^2 u_m - I_{ion}^m + 2\frac{D_z}{d_z^2}\chi_{m+1}(u_b - u_m)\\
\partial_t u_b &= \alpha D_o\nabla_\perp^2 u_b - I_{ion}^b + \alpha\frac{D_z}{d_z^2}(1-\chi_{b+1})(u_m - u_b) + \alpha\frac{D_z}{d_z^2}(u_{b-1} - u_b)\\
\partial_t u_{b-1} &= \alpha D_o\nabla_\perp^2 u_{b-1} - I_{ion}^{b-1} + \alpha\frac{D_z}{d_z^2}(u_b - u_{b-1}),
\end{aligned} \tag{13}$$

where the subscript $b-1$ refers to the transmembrane voltage and ionic currents in the extra boundary slice. Notice that the first equation in (13) is equivalent to the case $l = d_z$, while the second equation has the extra term $\alpha D_z(u_{b-1} - u_b)/d_z^2$.

Although Eq. (13) is already compact, we ask ourselves if a 2-slice model can approximate the 3-slice model in order to gain mechanistic insights on how the size of the boundary layer affects the boundary-driven feedback. To

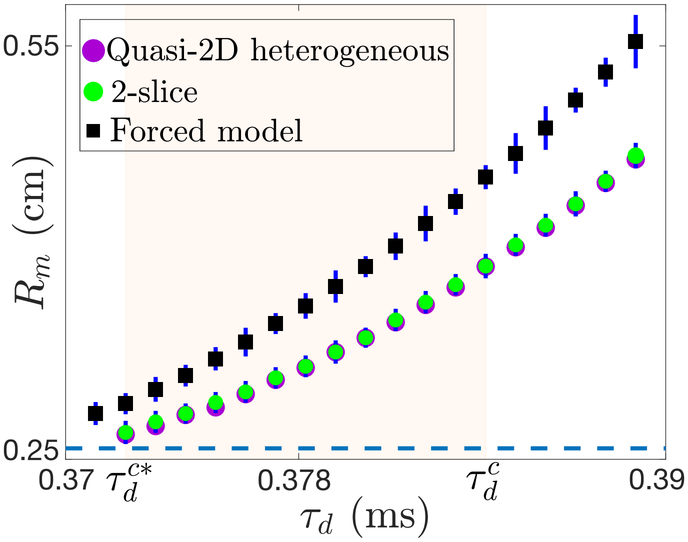

Figure S6: Comparison of the core radius between the full heterogeneous model in a tissue slab of thickness $\mathcal{H} = 14d_z$, with boundary heterogeneities of strength $\alpha = 0.005$ and size d_z, the 2-slice model, and the forced model. Blue bars indicate standard deviation associated with errors in the tip detection algorithm. The dashed lines correspond to the critical R_m value in the homogeneous case.

address this question, it would be necessary to write u_{b-1} as a function of u_b and u_m. Inspired by our findings in the main text, we envision that a good approximation is $u_{b-1} = u_m + q(u_b - u_m)$. We estimate q by calculating the ratio $(u_{b-1} - u_m)/(u_b - u_m)$ from numerical integrations of the full quasi-2D equations (1-8). A priori, q is not a constant and it has $x-y$ dependency, which is a combination of numerical error from the model discretization and finite spatial gradients. We decide to only extract the ratio information close to the wave front of the spiral wave in the middle slice (Fig. S7A). This is motivated by the observation that the mismatch of transmembrane voltages at the bulk-boundary layer interface is stronger at the wave front (see the bottom panel of Fig. 3D in the main text). Finally, we take the spatial and temporal (over $15T_{rot}$) average of the numerical ratio, obtaining q. Then, in particular, we can rewrite the equation for u_b:

$$\partial_t u_b \approx \alpha D_o \nabla_\perp^2 u_b - I_{ion}^b + \alpha \frac{D_z}{d_z^2}(2 - \chi_{b+1} - q)(u_m - u_b). \tag{14}$$

Numerically integrating the above equation together with the first equation in (13) yields the excellent agreement shown in Fig. 4B of the main text for $\mathcal{H} = 14d_z$, $\alpha = 0.005$, and $q = 1.17$. This agreement indicates that q, computed only at τ_d^o, depends solely on geometrical factors (h, l, d_z) and on α, similar to χ_k.

The reduction from a 3-slice to a 2-slice model allows the derivation of a forced model as in the main text. In fact, this new forced model is equivalent to Eq. (3) in the main text (corresponding to $l = d_z$), except that the time scale $\mathcal{T}_{bl}$ must be redefined as $\mathcal{T}_{bl}^{-1} = \alpha D_z(2 - \chi_{b+1} - q)/d_z^2$. Note that χ_{b+1} and χ_{m+1}, the latter controlling $\mathcal{T}_b$, are updated through their dependence on h and l. Integration of the forced model reproduces reasonably well the enhancement of stability observed at $l = 2d_z$ (Fig. 4B in the main text), which exceeds that for $l = d_z$. This increase in $\Delta\tau_d^c$ for larger boundary layers correlates with a decrease in the ratio $\mathcal{T}_b/\mathcal{T}_{bl}$ (Fig. S7B), indicating that thicker boundary layers produce slower boundary feedback relative to bulk excitability loss.

XI. Control of spiral wave break-up through boundary layer heterogeneities

The stabilizing properties of boundary layer heterogeneities are not limited to the meandering instability but can be extended to spiral wave break-up. To illustrate this, we modify the electrophysiological parameters (Table S1) in the FK model and use set 4 from Ref. [10] such that a rigidly rotating spiral wave in homogeneous tissue undergoes break-up. As detailed in this reference, this break-up is driven by discordant alternans when τ_d is smaller than a critical value $\tau_d^{c,SDC} = 0.404$ ms (Fig. S8A). We use the computationally efficient Eq. (2) from the main text, which offers a speed-up that can be estimated to be $(\mathcal{H} + d_z)/(2d_z)$, to implement the boundary layer effects, but we have verified that the full quasi-2D equations give similar results. As in the main text, we start with a stable spiral wave and a parameter value larger than the critical value ($\tau_d = 0.415$ ms) and decrease it in steps of 0.001 ms every $30T_{rot}$. We use a bigger domain size, $L = 12.24$ cm, in order to have sufficient space for the development of the break-up instability. The simulations reveal that the introduction of a boundary layer can enhance the stability of the spiral

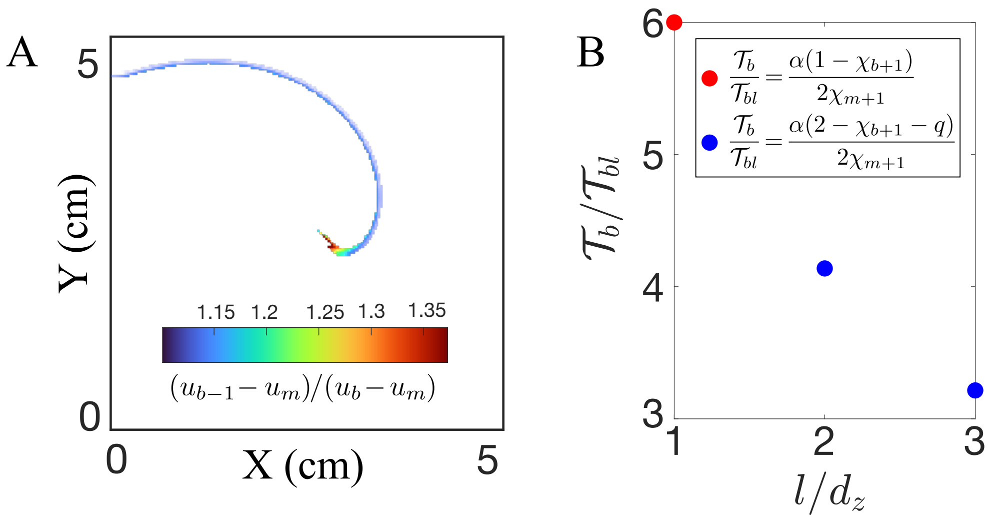

Figure S7: Modeling larger boundary layer effects in a slab of thickness $\mathcal{H} = 14d_z$ and heterogeneities of strength $\alpha = 0.005$. (A) A temporal snapshot of the numerical ratio $(u_{b-1} - u_m)/(u_b - u_m)$ near the wave front. (B) The dependence of $\mathcal{T}_b/\mathcal{T}_{bl}$ on the size of the boundary layer. In the case $l = 3d_z$, $q = 1.19$.

wave and prevents the break-up initiation. An example is shown in Fig. S8A, which shows the stabilization in the case of $\alpha = 0.0002$ and $h = 12d_z$ ($\mathcal{H} = 14d_z$). Instead of breaking up into multiple small-scale excitations, the spiral wave remains stable and displays a circular trajectory.

As in the main text, we perform a systematic variation of the thickness and the coupling strength, and construct a phase diagram showing the enhancement of stability in the break-up scenario $\Delta\tau_d^{c,SDC}/\tau_d^{c,SDC}$ (Fig. S8B). Interestingly, when $\mathcal{H}/d_z > 4$, the strongest stabilization is observed in the leakage regime ($\alpha < \alpha_f$), different from the behavior in the meandering scenario. In this same regime, but when $\mathcal{H}/d_z \leq 4$, we observe that spiral waves do not break up when sweeping down τ_d; instead, they undergo meandering instabilities.

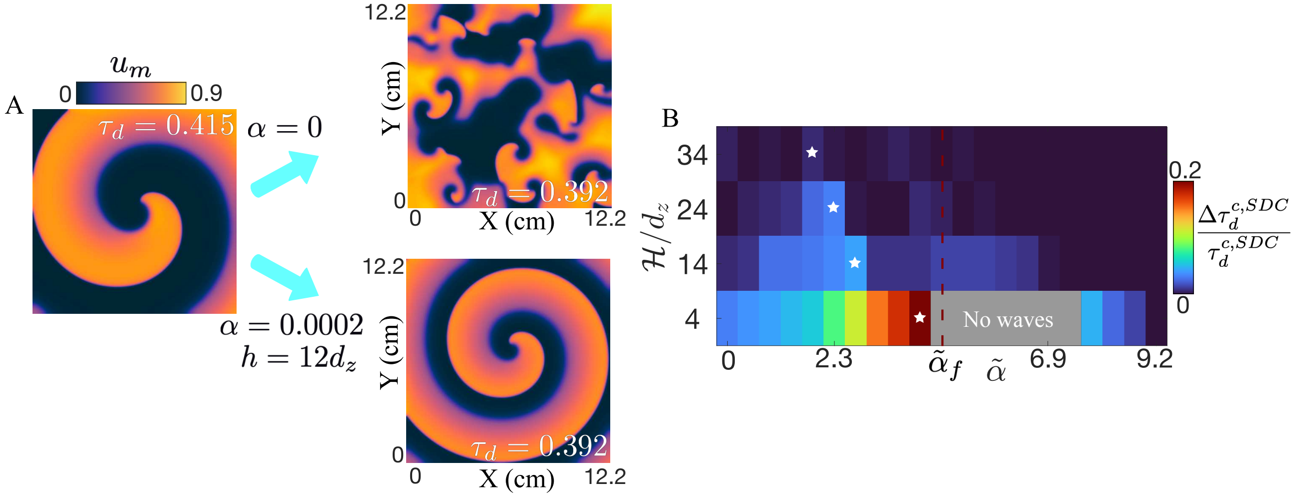

Figure S8: Control of spiral wave break-up through boundary layers of size d_z. (A) Spiral wave dynamics following a reduction of τ_d below the critical value in the homogeneous case (left panel) and in the presence of a boundary layer heterogeneity (right panel). (B) Phase diagram in $\tilde{\alpha} - \mathcal{H}/d_z$ space showing the level of enhancement of stability in the break-up case $\Delta\tau_d^{c,SDC}/\tau_d^{c,SDC}$. Gray region corresponds to parameter values for which wave activity is terminated. The white stars indicate $\max(\Delta\tau_d^c/\tau_d^c)$ for each slab thickness.

XII. Reduced excitability of boundary layer cells

In the main text, we model boundary layer heterogeneities using a reduction in the coupling strength of cardiomyocytes. An alternative and equally valid approach is to model boundary layer heterogeneities as a decrease in the excitability of cells. In our modeling framework, this can be easily implemented by decreasing τ_r^0 in the boundary

layer from its bulk value $\tau_r = 33$ ms. We have verified that in this case the model still displays an enhancement of stability against the meandering instability, even for the case $\alpha = 1$ (i.e., no heterogeneity in the coupling strength). For example, for $\tau_r^0 = 10$ ms, we find $\Delta\tau_d^c/\tau_d^c = 0.094$ in a tissue slab of thickness $\mathcal{H} = 14d_z$ and a boundary layer of size $l = d_z$. This enhancement is also present for $\alpha < 1$, corresponding to a boundary heterogeneity in both the coupling strength and in the ionic current I_{so} (red circles, Fig. S9). Note that the enhancement of stability in this case is significantly increased when compared to the case addressed in the paper (blue circles, Fig. S9). Obviously, this enhancement depends on the degree of excitability reduction, parameterized by τ_r^0 (see below). Altogether, the equivalence in wave stabilization despite the type of heterogeneity can be qualitatively explained by the fact that boundary layer effects become important when the characteristic length of the wave is $\mathcal{O}(l)$. This length can be tuned by varying either the coupling strength or the time scales in the local dynamics of the transmembrane voltage.

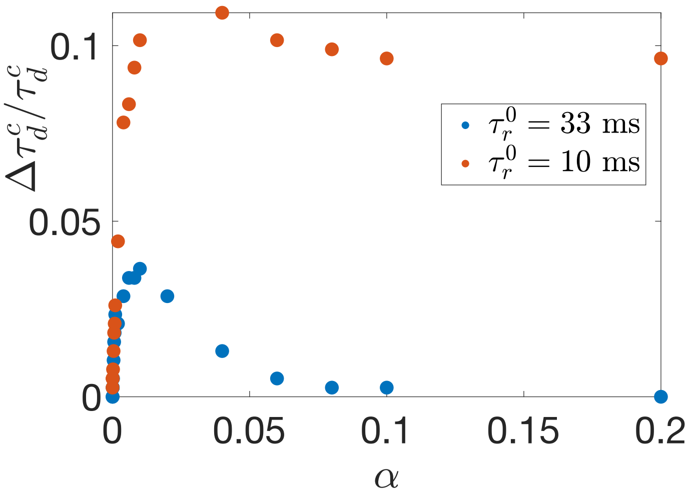

Figure S9: Enhancement of stability as a function of α in a tissue slab of thickness $\mathcal{H} = 14d_z$ with a boundary layer of size $l = d_z$ and with $\tau_r = 33$ ms in the bulk. Plotted is the enhancement in the presence of reduced excitability in the boundary layer ($\tau_r^0 = 10$ ms; red circles) and for the case of ionic homogeneity ($\tau_r^0 = 33$ ms; blue circles). Note that for $\alpha > 0.2$, the enhancement saturates at $\Delta\tau_d^c/\tau_d^c = 0.094$ in the presence of the ionic heterogeneity and that wave activity terminates between $\alpha = 0.01$ and $\alpha = 0.04$.

For completeness, we have verified that reducing the excitability within the boundary layers, while keeping $\alpha = 1$, can also suppress break-up instabilities driven by discordant alternans (Fig. S10). Here, the reduction is introduced via $\tau_r^0 = \beta\tau_r$, with β a constant, to characterize how the enhancement of stability depends on the degree of excitability reduction. In this case, $\Delta\tau_d^c/\tau_d^c$ increases monotonically as β decreases, until the excitability reduction is strong enough to suppress all wave activity (Fig. S10). Thus, and in contrast to the case $\alpha = 0$ and $\alpha = 1$, choosing $\beta = 0$ or $\beta = 1$ does not result in equivalent results. Finally, larger boundary layers strengthen the stabilization, similar to the case of meandering.

XIII. Discretization and propagation failure on the $x-y$ plane

In this section, we explore the effects of varying Δx on the suppression of meandering instabilities. Specifically, we make the in-plane spatial grid finer, from $\approx 10d_z$ to $\sqrt{5}d_z$. This particular choice results in equal conduction strength in the $x-y$ and transmural directions. Similar to the analysis in the main text, we sweep the excitability parameter τ_d, in steps of 0.001 ms, starting from a rigidly rotating scroll wave and measure the corresponding enhancement of stability $(\tau_d^{c*} - \tau_d^c)/\tau_d^c = \Delta\tau_d^c/\tau_d^c$. The results show that the difference is negligible and at most equal to the discretization step in τ_d (Fig. S11). This plot shows that the propagation failure along the transmural direction is independent of Δx and occurs at $\alpha = \alpha_f$. Note, however, that the propagation failure in the $x-y$ plane within the boundary layer depends on the discretization and occurs at different α_f^l values (Fig. S11). Therefore, our results suggest that the stabilization mechanism found in the main text is governed by transmural dynamics.

XIV. Numerical implementation of the quasi-2D model in WebGL

Following recent efforts to develop GPU-accelerated cardiac research codes that can be easily used on personal laptops [11, 12], we created a WebGL implementation of the quasi-2D Fenton-Karma model studied in this work [2]. Specifically, we combined the libraries Abubu.js and CCapture.js to build a GPU-accelerated code for 3D simulations in a simple geometry, capable of storing data while bypassing the usual GPU-CPU memory-transfer bottleneck. This storage protocol is possible because the WebGL simulations run on a web browser (e.g., Google Chrome, Safari, Firefox), which enables the direct implementation of an algorithm that simply takes screenshots of a canvas on the webpage. To fully exploit this capability, we solved the three-dimensional equations in an extended two-dimensional

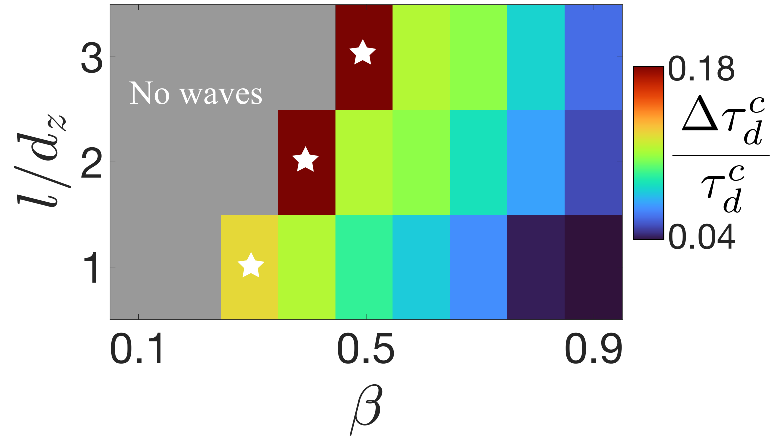

Figure S10: Effects of excitability reduction, parameterized by β, and boundary layer size l in the enhancement of scroll wave stability $\Delta\tau_d^c/\tau_d^c$ in the wave break-up regime. The white stars indicate $\max(\Delta\tau_d^c/\tau_d^c)$ for each boundary layer size.

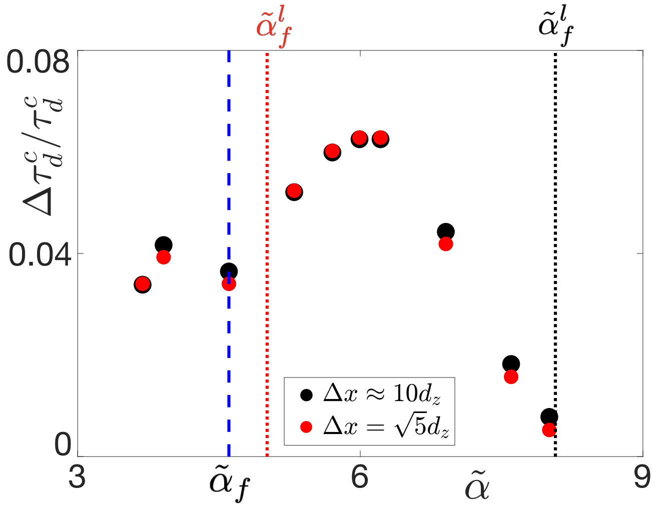

Figure S11: Effects of Δx in the enhancement of stability of scroll waves in a slab of thickness $\mathcal{H} = 8d_z$ with boundary layers of size $l = d_z$. The dashed blue line indicates propagation failure along the transmural direction, while the dotted lines represent propagation failure in the $x-y$ plane. The α_f^l values were obtained from 2D simulations in a single boundary slice. As in the main text, $\tilde{\alpha} = log(\alpha) + 10.82$.

domain: the z-direction is mapped onto the x-direction (Fig. S12). Thus, discrete differences such as $u_{i,j,k+1} - u_{i,j,k}$ were encoded as $u_{i+L/\Delta x+1,j} - u_{i,j}$, where i (j) indexes the discretization of the $x(y)$-direction.

The algorithm begins with an initial condition in RGBA format, created in Matlab, containing the transmembrane voltage u and gating variables v and w encoded in the RGB channels, respectively (Fig. S12A). The transparency channel encodes the distribution of heterogeneities, $\mathcal{I}_k$, as well as the limits between z-slices in the extended domain, where non-flux boundary conditions are imposed in the x and y directions. Then, the WebGL code integrates Eqs. (1)-(8) while displaying the u field in real time on one canvas (Fig. S12B). At the same time, a numerical algorithm that computes the spiral tip position in each z-slice updates a second canvas (Fig. S12C). Finally, the stored data is processed using Matlab pipelines [2].

XV. Video caption

Video S1: Scroll wave stabilization through boundary layer heterogeneities in a slab of thickness $\mathcal{H} = 14d_z$. The video shows the spiral tip trajectories (red circles) on the bulk slices bordering the boundary layers. Initially, the scroll wave exhibits meandering, but it stabilizes after the instantaneous decrease of α from 1 to 0.004 at time $t_{het} = 0.2$ s. The stabilization suppresses the meandering instability, and the scroll wave enters a rigidly rotating regime.

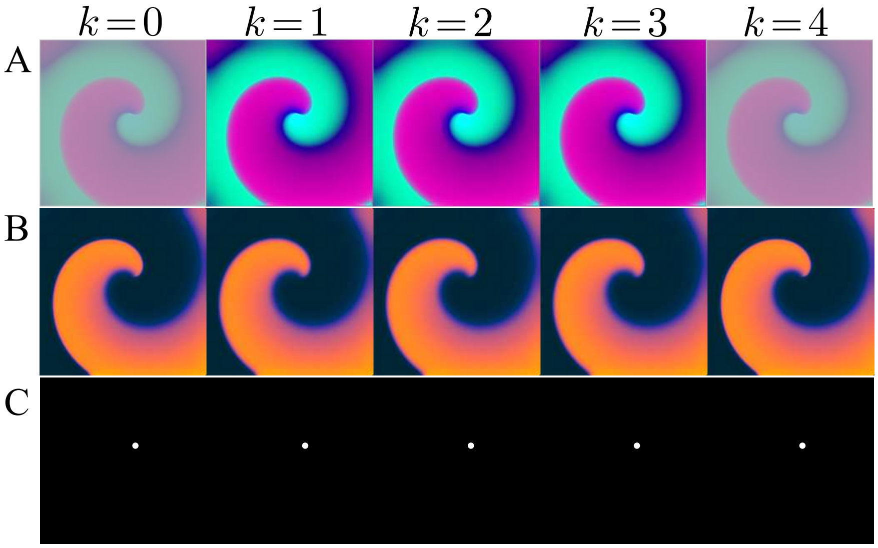

Figure S12: WebGL example for the case $\mathcal{H} = 4d_z$ and $l = d_z$. (A) Initial condition in RGBA format with the $\{u, v, w\}$ fields, the $\mathcal{I}_k$ distribution, and the non-flux boundary information. (B) Canvas showing the u field for $\alpha = 0.02$ and $\tau_d = 0.38$ ms. (C) Spiral tip positions of the waves in (B), enlarged for visualization purposes.

References

[1] F. Fenton and A. Karma, Chaos: An Interdisciplinary Journal of Nonlinear Science **8**, 20 (1998).
[2] S. Echeverria-Alar, 10.5281/zenodo.17718759 (2025).
[3] W.-J. Rappel, Physics reports **978**, 1 (2022).
[4] W.-J. Rappel, Chaos: An Interdisciplinary Journal of Nonlinear Science **11**, 71 (2001).
[5] S. Pravdin, H. Dierckx, V. S. Markhasin, and A. V. Panfilov, BioMed research international **2015**, 389830 (2015).
[6] F. H. Fenton, E. M. Cherry, A. Karma, and W.-J. Rappel, Chaos: An Interdisciplinary Journal of Nonlinear Science **15** (2005).
[7] C. B. Macdonald, B. Merriman, and S. J. Ruuth, Proceedings of the National Academy of Sciences **110**, 9209 (2013).
[8] B. Sandstede and A. Scheel, Spiral waves: linear and nonlinear theory, 1413 (American Mathematical Society, 2023).
[9] H. Henry and V. Hakim, Physical Review E **65**, 046235 (2002).
[10] F. H. Fenton, E. M. Cherry, H. M. Hastings, and S. J. Evans, Chaos: An Interdisciplinary Journal of Nonlinear Science **12**, 852 (2002).
[11] A. Kaboudian, E. M. Cherry, and F. H. Fenton, Science advances **5**, eaav6019 (2019).
[12] A. Kaboudian, R. A. Gray, I. Uzelac, E. M. Cherry, and F. H. Fenton, Computer Methods and Programs in Biomedicine **257**, 108456 (2024).
